# The study of *METTL3* and *METTL14* expressions in childhood *ETV6/RUNX1*‐positive acute lymphoblastic leukemia

**DOI:** 10.1002/mgg3.933

**Published:** 2019-08-20

**Authors:** Congcong Sun, Lixian Chang, Chao Liu, Xiaoyan Chen, Xiaofan Zhu

**Affiliations:** ^1^ Center for Pediatric Blood Disease, State Key Laboratory of Experimental Hematology, Institute of Hematology and Blood Diseases Hospital Chinese Academy of Medical Sciences and Peking Union Medical College Tianjin P.R. China

**Keywords:** acute lymphoblastic leukemia, children, *ETV6/RUNX1*, *METTL14*, *METTL3*

## Abstract

**Background:**

This study was aimed to explore the *METTL3* and *METTL14* expressions in children with *ETV6/RUNX1(E/R)*‐positive acute lymphoblastic leukemia (ALL) and investigate the relation between the *METTL3* and *METTL14* expressions with clinical features.

**Methods:**

Thirty‐seven newly diagnosed *E/R*‐positive ALL children and six controls were included in this study. Real‐time quantitative polymerase chain reaction (RT‐PCR) was used to detect the mRNA expression level of *METTL3* and *METTL14*.

**Results:**

Among the 37 cases, 51.35% (*n* = 19) were boys and 48.65% (*n* = 18) were girls and the median age was 4.72 (1.72–11.99) years. Among the six controls, 50% (*n* = 3) were boys and 50% (*n* = 3) were girls and the median age was 5.24 (1.53–13.17) years. The expression level of *METTL3* and *METTL14* in *E/R*‐positive ALL patients were lower than in controls (*p* < .05). Although failed to achieve statistical significance, the expression level of *METTL3* and *METTL14* in relapse patients were lower than nonrelapse patients (*p* = .171, *p* = .150, respectively).

**Conclusion:**

The reduced levels of *METTL3* and *METTL14* suggest a possible role in the pathogenesis and course of *E/R*‐positive ALL. *METTL3* and *METTL14* may become new prognostic factors, and rationalize specific treatment intensification in possible *E/R*‐positive relapse patients.

## 1 INTRODUCTION

The translocation t(12;21)(p13;q22) generating *ETV6/RUNX1* (TEL/AML1) fusion gene occurs in ~ 25% of childhood B‐cell precursor acute lymphoblastic leukemia (ALL) (Harbott, Viehmann, Borkhardt, Henze, & Lampert, 1997). The *ETV6/RUNX1*(*E/R*) fusion gene has been reported to originate in the prenatal period based on detecting the fusion sequence in identical twins and in neonatal blood spot of children with ALL (Zuna et al., 2011). The transformation of *E/R* fusion gene results in the generation of a persistent preleukemic clone, which postnatally converts, at low frequency, to ALL after the acquisition of necessary secondary genetic abnormalities (Papaemmanuil et al., 2014). These secondary genetic events are major rate‐limiting events during the process of formation of *E/R*‐positive ALL and may be expected to drive the evolution of overt leukemic cells, culminating in a clinical diagnosis of ALL. However, the mechanisms triggering these second hits have not been completely elucidated. In addition, although generally associated with favorable risk features and advantageous prognosis, relapses can occur in as many as 20% of patients (Gandemer et al., 2012). Most of the patients relapses several years after cessation of treatment (Forestier et al., 2008) and occasionally after 10–20 years (Chow, Dalla‐Pozza, Gottlieb, & Hertzberg, 1999).

Recent years, epigenetic modifications have been reported to contribute significantly to leukemogenesis (Busche et al., 2013). N6‐methyladenosine (m6A) is the most prevalent and reversible internal modification in mammalian messenger and noncoding RNAs (Liu et al., 2014). This modification can be installed by methyltransferase that serve as “writers” and can also be reversed by demethylases that serve as “erasers.” It is widely conserved among eukaryotic species that range from yeast, plants, and flies to mammals as well as among viral mRNAs (Yue, Liu, & He, 2015). *METTL3* (OMIMl: 612472) and *MTEEL14* (OMIMl: 616504) are two active components of the m6A methyltransferase complex in mammalian cells and can influence mRNA transcription, splicing, nuclear export, localization, translation, and stability (Fu, Dominissini, Rechavi, & He, 2014). So far, there is no study to characterize the m6A in childhood patients with ALL. To investigate whether m6A play a role in the emergence and development of leukemia, we performed real‐time fluorescent quantitative PCR to detect the mRNA expression level of *METTL3* and MTEEL14 in *E/R*‐positive ALL.

## 2 METHODS

### 2.1 Ethical compliance

Our study was approved by the ethics committee of Institute of Hematology and Blood Diseases Hospital, Chinese Academy of Medical Sciences (KT2015045‐EC‐2). Guardians of the patients and controls all signed the informed consent. From July 2007 to December 2014, 37 children with *E/R*‐positive ALL children in Institute of Hematology and Blood Disease Hospital were enrolled in our study. Chemotherapy protocol of ALL patients referred to CCCG‐ALL 2008 protocol. Two normal children and four immune thrombocytopenia (ITP) patients were also collected as normal controls. These *E/R*‐positive patients were divided into standard risk (SR), intermediate risk (IR), and high risk (HR) groups (referred to CCCG‐ALL 2008 protocol), the day 33 minimal residual disease (MRD)‐positive and negative groups, and relapse and nonrelapse groups. The characteristics of *E/R*‐positive patients are summarized in Table 1.

**Table 1:** Characteristics of *E/R*‐positive patients

Characteristics	*ETV6/RUNX1*(+) patients
Age, years, median (range)	4.72 (1.72–11.99)
Gender	
Male	19 (51.35%)
Female	18 (48.65%)
WBC (◊10^9^/L), median (range)	11.56 (1.6–253)
Blast in bone marrow median (%, range)	82.76 (31.55–95.5)
LDH median (U/L, range)	401 (199–1307)
Risk group	
SR	15 (40.54%)
IR	13 (35.13%)
HR	9 (24.33%)
D33 MRD	
D33 MRD (+)	26 (70.27%)
D33 MRD (−)	11 (29.73%)
Prognosis	
Nonrelapse	27 (72.97%)
Relapse	10 (27.03%)

Abbreviations: HR, high risk; IR, intermediate risk; MRD, minimal residual disease; SR, standard risk.

### 2.2 Isolation of leukemic cells from bone marrow

Five milliliters of bone marrow was collected from each patient and control at the time of diagnosis. Human mononuclear cells were separated from bone marrow using Ficoll‐Paque PLUS (GE HealthCare Life Sciences) according to the manufacturer's instructions. Then primitive lymphocytes were obtained by BD FACSAria Ⅲ flow cytometer by staining CD10 and CD19 antibody.

### 2.3 Quantitative real‐time polymerase chain reaction analysis

Total RNA was isolated from the leukemic cells using the QIAGEN RNeasy Mini Kit and used for cDNA synthesis by TransScript First‐Strand cDNA Synthesis SuperMix. RNA quality was analyzed by NanoDrop. *METTL3* (NM_019852.5) and *METTL14* (NM_201638.2) transcripts were quantified by SYBRw Green PCR kit using the ABI 7900HT Fast Real‐Time PCR Instrument (Applied Biosystems). The sequences of the amplification primers for *METTL3* and *METTL14* are listed in Table 2. The amplification efficiency between the target (i.e., *METTL3*) and the reference control (i.e., *GAPDH*) was compared to use the delta delta Ct (ΔΔCt) calculation.

**Table 2:** Primers and conditions for the real‐time PCR experiments performed in this study

Gene name		Primer sequence (5’→3’)	T (°C)	Product length
*METTL3*	F	ATGGGAAGGAACACTGCTTG	60.11	104 bp
	R	ATGACTGGTGGAACGAACCT		
*METTL14*	F	ATCGCCTCCTCCCAAATCTA	60.32	160 bp
	R	ACCTCTGTGTGCTCCTCCAC		
GAPDH	F	ACCCAGAAGACTGTGGATGG	59.96	125 bp
	R	TTCAGCTCAGGGATGACCTT		

### 2.4 Statistical analysis

The data are expressed as mean ± *SD*. All the data were analyzed by SPSS21.0 and GraphPad Prism5.0 software. Statistical significance between *E/R*‐positive patients and controls and different groups were assessed by the paired two‐tailed *t* tests. Correlation analysis was assessed by means of Pearson correlation analysis. *p*‐values ≤ .05 were considered statistically significant.

## 3 RESULTS

### 3.1 *METTL3* and *METTL14* expressions in *E/R*‐positive patients and controls

**Figure 1:**
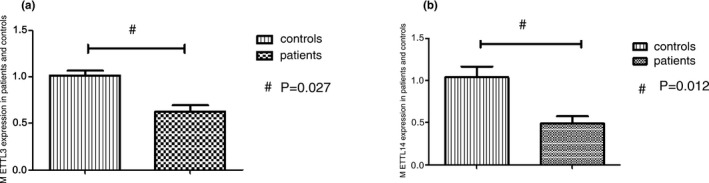
*METTL3* and *METTL14* expressions in *E/R*‐positive patients and controls. (a) The relative levels of mRNA gene expression of *METTL3* were decreased in *E/R*‐positive patients compared with controls, ^#^ *p* = .027. (b) The relative levels of mRNA gene expression of *METTL14* were decreased in *E/R*‐positive patients compared with controls, ^#^ *p* = .012

The data are presented as fold changes in gene expression normalized to an endogenous reference gene and relative to controls. The relative levels of mRNA gene expression of *METTL3* were decreased 0.61‐fold (*p* < .05) in *E/R*‐positive patients compared with controls (Figure 1a). The decreases observed in *METTL14* mRNA expression were 0.47‐fold (*p* < .05) in *E/R*‐positive patients compared with controls (Figure 1b). In addition, the gene expression level of *METTL3* and *METTL14* was correlated (Pearson r = .863, r^2^ = .745, *p* < .01).

### 3.2 *METTL3* and *METTL14* expressions in different risk groups of patients

**Figure 2:**
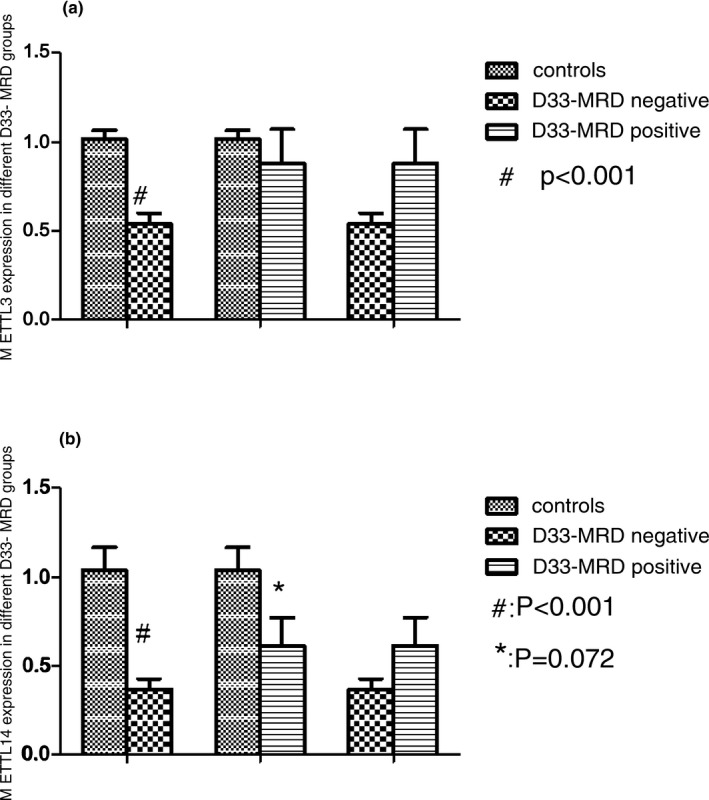
*METTL3* and *METTL14* expressions in different risk groups. (a) The gene expression of *METTL3* in SR, IR, and HR were lower than in controls. ^#^ *p* < .001, patients (SR) versus normal controls, *p = .002, patients (HR) versus normal controls. (b) The gene expression of *METTL14* in SR, IR, and HR were lower than in controls. ^#^ *p* < .001, patients (SR) versus normal controls, *p = .05, patients (IR) versus normal controls. ^&^ *p* < .001, patients (HR) versus normal controls. Abbreviations: HR, high risk; IR, intermediate risk; SR, standard risk

The gene expression of *METTL3* in SR, IR, and HR were lower than in controls. The relative levels of mRNA gene expression of *METTL3* were decreased 0.52‐fold, 0.85‐fold, and 0.54‐fold in SR, IR, and HR groups respectively compared with controls. Except for IR group, differences of the gene expression in SR and HR all reached statistical significances (Figure 2a). Compared with controls, the gene expression of *METTL14* in SR, IR, and HR groups were decreased 0.38‐fold, 0.57‐fold, and 0.35‐fold, respectively and all had statistical significances (Figure 2b). However, the gene expression of *METTL3* and *METTL14* did not have statistical significances between three risk groups.

### 3.3 *METTL3* and *METTL14* expressions in D33 MRD‐positive and negative groups

**Figure 3:**
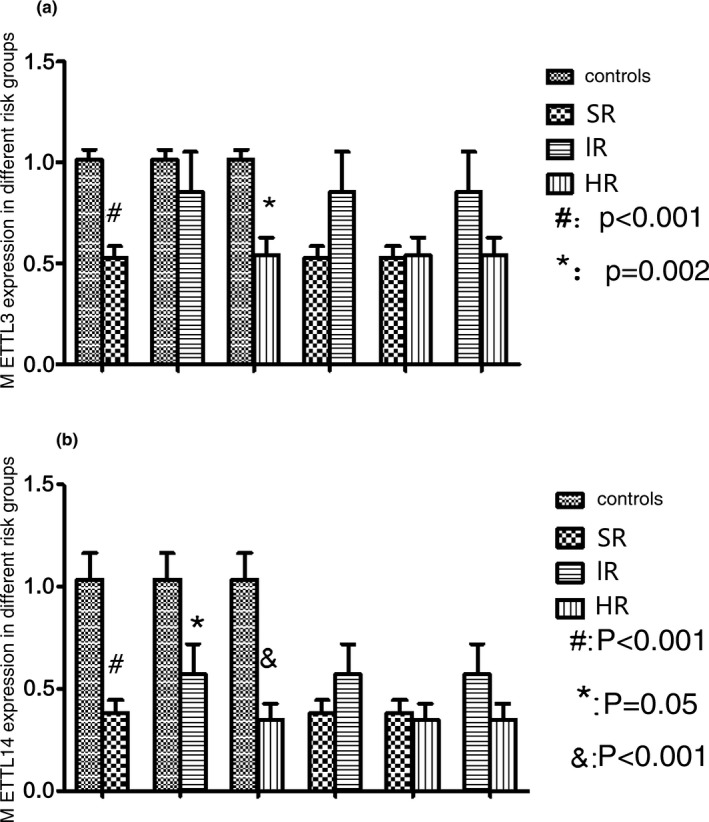
*METTL3* and *METTL14* expressions in different D33 MRD groups. (a) The gene expression of *METTL3* was decreased in D33 MRD‐negative and positive groups compared with controls. ^#^ *p* < .001, patients (D33 MRD‐negative) versus normal controls. (b) The gene expression of *METTL14* was decreased in D33 MRD‐negative and positive groups compared with controls. ^#^ *p* < .001, patients (D33 MRD‐negative) versus normal controls. ^*^ *p* = .072, patients (D33 MRD‐positive) versus normal controls. Abbreviation: MRD, minimal residual disease

The gene expression of *METTL3* was decreased 0.58‐fold (*p* < .05) and 0.85‐fold (*p* > .05) in D33 MRD‐negative and positive groups compared with controls (Figure 3a). The decreases observed in the relative levels of mRNA gene expression of *METTL14* were 0.36‐fold (*p* < .05) and 0.61‐fold (*p* < .05) in D33 MRD‐negative and positive groups compared with controls (Figure 3b). Statistical difference was not found in the D33 MRD‐negative patients compared to the D33 MRD‐positive patients (*p* > .05).

### 3.4 The *METTL3* and *METTL14* expressions in relapse and nonrelapse patients

**Figure 4:**
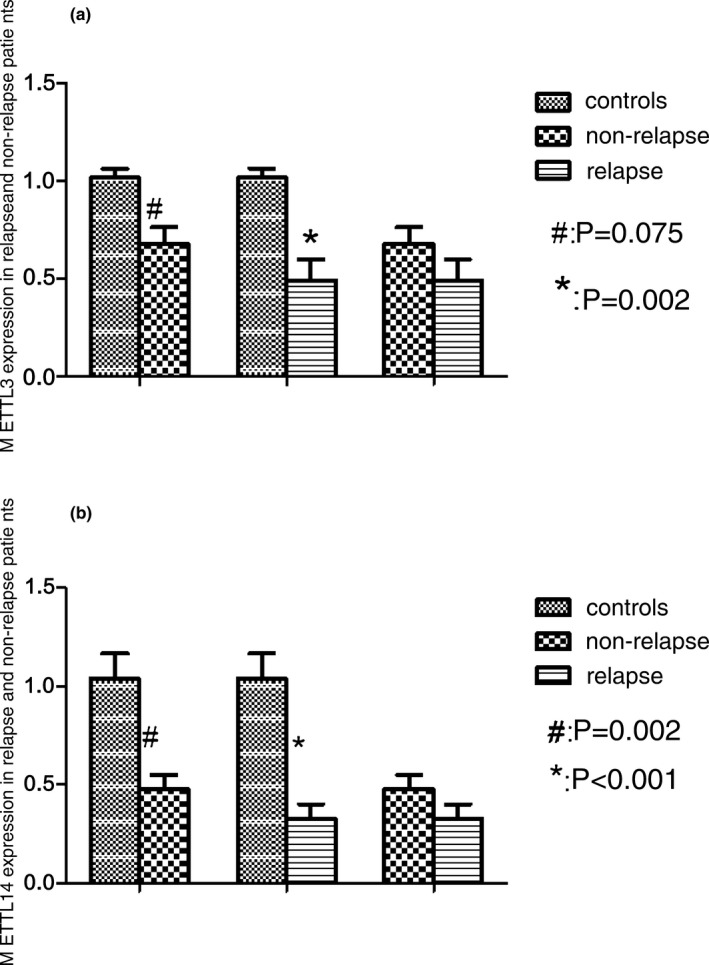
*METTL3* and *METTL14* expressions in relapse and nonrelapse patients. (a) The relative levels of mRNA gene expression of *METTL3* were decreased in nonrelapse and relapse patients compared with controls, respectively. ^#^ *p* = .075, nonrelapse patients versus normal controls. *p = .002, relapse patients versus normal controls. (b) The relative levels of mRNA gene expression of *METTL14* were decreased in nonrelapse and relapse patients compared with controls, respectively. ^#^ *p* = .002, nonrelapse patients versus normal controls. *p < .001, relapse patients versus normal controls

The relative levels of mRNA gene expression of *METTL3* were decreased 0.67‐fold (*p* < .05) and 0.49‐fold (*p* < .05) in nonrelapse and relapse patients compared with controls, respectively (Figure 4a). *METTL14* expression were decreased 0.47‐fold (*p* < .05) and 0.32‐fold (*p* < .05) in nonrelapse and relapse patients compared with controls (Figure 4b). Although did not achieve statistical significance, the gene expression of *METTL3* and *METTL14* was lower in relapse patients than nonrelapse patients.

### 3.5 The correlation of gene expression of *METTL3* and *METTL14* with clinical data

There was no correlation between the expression level of *METTL3* and *METTL14* with gender, age, initial white blood cell count, blast cells percentage, and the level of LDH (*p* > .05).

## 4 DISCUSSION

In pediatric B‐cell ALL, the *E/R* fusion gene is the most frequent chromosomal lesion (Romana et al., 1995; Shurtleff et al., 1995). This alteration occurs in approximately 25% of childhood ALL diagnosed between the ages of 2 and 10 years, with a median age of 4 years (Golub, Barker, Stegmaier, & Gilliland, 1996; Harbott et al., 1997; McLean et al., 1996). Thirty‐seven *E/R*‐positive ALL children were enrolled in our study, with a median age of 4.72 years, which was in accordance with the published data. Based on the excellent molecular response to treatment and beneficial clinical outcome, it was originally believed that this rearrangement is a rather favorable prognostic indicator (Rubnitz et al., 1999; Uckun et al., 2001). However, this notion was subsequently disputed as others found predominantly late relapses occurring in up to 20% of patients (Harbott et al., 1997; Seeger et al., 1998). In our study, 10 out of 37 patients developed relapse, with a ratio of 27.03%. Thus, during the past several years, investigators have focused on deciphering the events required to develop *E/R*‐positive leukemia and relapse.

Epigenetic alterations, including DNA methylation and histone modifications, have been reported to contribute to ALL progression (Chen, Odenike, & Rowley, 2010; Milani et al., 2010). Stephan et al combined methylome and transcriptome approaches to identify epigenetic biomarkers specific for *E/R*‐positive ALL, and showed the effects of methylation on the expression of 17 potential drivers of leukemogenesis (Busche et al., 2013). Recent years, N6‐methyladenosine (m6A) is the most prevalent and reversible internal modification in mammalian messenger and noncoding RNAs (Cantara et al., 2011; Liu et al., 2014). *METTL3* and *METTL14* are components of m6A‐methyltransferase complex that actively involved in the posttranscriptional methylation of internal adenosine residues (Wu, Jiang, Wang, & Wang, 2016). *METTL14* shares 43% identity with *METTL3* and is demonstrated as a homolog of *METTL3* (Bujnicki, Feder, Radlinska, & Blumenthal, 2002). Ping Wang et al have verified that the *METTL3* is the catalytically active subunit while *METTL14* plays a structural role critical to substrate recognition (Wang, Doxtader, & Nam, 2016). Xiao‐Li Ping et al confirmed the interaction between *METTL3* and *METTL14* by using co‐immunoprecipitation (Ping et al., 2014). In our study, the gene expression of *METTL3* and *METTL14* had a strong correlation (Pearson r = .863, r^2^ = .745, *p* < .01), further demonstrating the synergistic effect of these two genes.

In our study, the gene expression of *METTL3* and *METTL14* were lower in *E/R*‐positive patients than in normal controls (*p* < .05). But there were no differences of the expression of these two genes in different risk groups and different D33 MRD groups (*p* > .05). Various studies have demonstrated that m6A manipulation via knockdown or deletion of the methyltransferase or demethylase can impact diverse biological functions, such as body mass and metabolism, synaptic signaling, circadian clock regulation, early embryonic development, and stem cell self renewal and differentiation (Lin, Choe, Du, Triboulet, & Gregory, 2016; Lin & Gregory, 2014; Merkestein et al., 2015). But its role in cancer has not been well studied. In a recent study, *METTL14* and *METTL3* have been demonstrated to be downregulation in hepatocellular carcinoma (Ma et al., 2017). This is consistent with our results. So we speculated that the downexpression of *METTL3* and *METTL14* in *E/R*‐positive ALL may affect the m6A modification of some genes in leukemic cells, and then promote the development of leukemia. However, in another study on lung adenocarcinoma, *METTL3* expression was elevated and could promote growth, survival, and invasion of human lung cancer cells (Lin et al., 2016). This phenomenon can be explained by the theory that methylation marks can be dynamically regulated and m6A patterns can vary between cell types (Geula et al., 2015; Meyer et al., 2012).

Although the gene expression differences of *METTL3* and *METTL14* between relapse and nonrelapse groups failed to achieve statistical significance, the expression level of these two genes were lower in relapse patients (*p* = .171, .150 respectively). A larger sample size may achieve statistical significance. Relapsed childhood *E/R*‐positive ALL is a clinically and biologically heterogeneous disease. Some studies compared diagnostic and relapse gene patterns to gain further insights into the molecular mechanisms of disease recurrence in *E/R*‐positive ALL (Bokemeyer et al., 2014; Grausenburger et al., 2015; Kuster, 2011). They found some recurrent genes such as *ETV6*, *BCL2L14*, and *CDKN1B* in relapsed *E/R*‐positive patients (Bokemeyer et al., 2014). Despite the research progress, the mechanism of leukemia relapse has not been fully elucidated. In a recent study, the downexpression of *METTL14* has been reported to act as an adverse prognosis factor for recurrence‐free survival of hepatocellular carcinoma and be associated with tumor metastasis in vitro and in vivo (Ma et al., 2017). Thus we speculate that the downexpression of *METTL3* and *METTL14* contributes to not only the development of leukemia but also to relapse. Additionally, we did not find any correlation between the expression level of *METTL3* and *METTL14* with gender, age, initial white blood cell count, blast percentage, and the level of LDH, indicating that these two genes may not be associated with tumor burden.

In conclusion, the expression level of *METTL3* and *METTL14* was much lower in *E/R*‐positive ALL patients than in controls and much lower in relapse patients than in nonrelapse patients. Thus, *METTL3* and *METTL14* may play important roles in the pathogenesis and relapse mechanism of pediatric *E/R*‐positive ALL patients. Further research on the precise role of these two genes in leukemogenesis and leukemia relapse are required. *METTL3* and *METTL14* may become new prognostic factors, and rationalize specific treatment intensification in possible *E/R*‐positive relapse patients.

## CONFLICT OF INTEREST

The authors declare that they have no competing interests.

## AUTHOR CONTRIBUTIONS

SCC, CLX, and ZXF designed the study and prepared the manuscript; SCC and LC involved in data collection; CXY performed data analysis.
